# Systems biology approaches for advancing the discovery of effective drug combinations

**DOI:** 10.1186/s13321-015-0055-9

**Published:** 2015-02-26

**Authors:** Karen A Ryall, Aik Choon Tan

**Affiliations:** Translational Bioinformatics and Cancer Systems Biology Laboratory, Division of Medical Oncology, Department of Medicine, School of Medicine, University of Colorado Anschutz Medical Campus, 12801 E.17th Ave., L18-8116, Aurora, CO 80045 USA; Department of Biostatistics and Informatics, Colorado School of Public Health, University of Colorado Anschutz Medical Campus, Aurora, CO USA; Department of Computer Science and Engineering, Korea University, Seoul, South Korea

**Keywords:** Drug combinations, Systems biology, Computational modeling, Cancer, Drug discovery

## Abstract

Complex diseases like cancer are regulated by large, interconnected networks with many pathways affecting cell proliferation, invasion, and drug resistance. However, current cancer therapy predominantly relies on the reductionist approach of one gene-one disease. Combinations of drugs may overcome drug resistance by limiting mutations and induction of escape pathways, but given the enormous number of possible drug combinations, strategies to reduce the search space and prioritize experiments are needed. In this review, we focus on the use of computational modeling, bioinformatics and high-throughput experimental methods for discovery of drug combinations. We highlight cutting-edge systems approaches, including large-scale modeling of cell signaling networks, network motif analysis, statistical association-based models, identifying correlations in gene signatures, functional genomics, and high-throughput combination screens. We also present a list of publicly available data and resources to aid in discovery of drug combinations. Integration of these systems approaches will enable faster discovery and translation of clinically relevant drug combinations.

**Graphical abstract:**
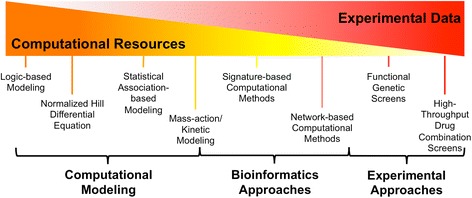
Spectrum of Systems Biology Approaches for Drug Combinations.

Spectrum of Systems Biology Approaches for Drug Combinations.

## Introduction

Despite increasing investments in pharmaceutical research and development, the rate of introduction of successfully translated drugs has decreased [1]. Reasons for increased attrition rates in drug development include toxicity and inadequate efficacy due to individual variation in therapeutic response and development of drug resistance [2]. This increased attrition rate has coincided with increased interest in seeking highly specific ligands affecting single targets for treatment of disease [3]. Pharmaceutical research has increasingly relied on reductionist approaches, even though systemic diseases such as cancer and heart disease are managed by large, interconnected networks with many pathways affecting pathological signaling [4,5]. The redundancy and feedback in these networks allows for robustness of phenotype and maintenance of homeostasis [6,7]. This network complexity has hindered development of new therapies and indicates a need for more integrative systems approaches to make better predictions of drug responses [8,9].

The failure of single targets to successfully translate into clinical practice and the problem of development of drug resistance with single target cancer therapies has increased interest in discovery of effective drug combinations. Administering drug combinations has been effective in overcoming resistance to anti-microbial therapies for treatment of infectious diseases such as HIV and tuberculosis [10]. In cancer, drug resistance can occur through mutation of the drug target [11], amplification of an alternate pathway [12], or intrinsic resistance of a subset of the cancer cells [13]. Combinations of drugs could potentially overcome these resistance strategies by limiting the potential of escape mutations and pathways [14].

**Figure 1:**
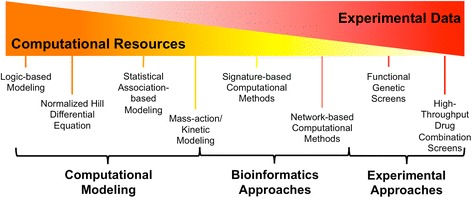
**Diagram depicting estimated ratio of computational and experimental requirements for various methods in this review.** For example, mass-action/kinetic modeling has higher experimental requirements than logic-based and normalized-Hill-based modeling due to its need for many abundance and rate parameters. Unbiased high-throughput screening of drug combinations has the highest experimental requirement. Many of the systems biology methods in this review aim to use publicly available data and computational approaches to reduce the need for exhaustive screens and prioritize combinations for experimental validation.

While combination therapies may dramatically improve efficacy of cancer therapies, the discovery of effective combinations is a challenging endeavor. With over 1,500 FDA approved compounds, experimentally testing every possible combination of these drugs would be unfeasible, even with high-throughput experimental methods [15]. Therefore, new systems approaches are needed to reduce the search space and prioritize combinations for experimental testing (Figure 1) [16].

## Review

Here, we review computational and experimental methods for accelerating the discovery of effective drug combinations for complex diseases, with special focus on cancer. In addition, we include a list of publicly available resources as a reference for future drug combination studies (Table 1).

**Table 1:** **Resources for systems analysis of drug combinations**

**Application**	**Resource**	**Description**	**URL**
**Drug data**	PubChem	Database of biological activities of millions of small molecules.	http://pubchem.ncbi.nlm.nih.gov/
	DrugBank	Database of target, chemical, pharmacological, and interaction data for 7739 drugs.	www.drugbank.ca/
	STITCH	Chemical-protein interaction database containing 300,000 small molecules and 2.6 million proteins from 1133 organisms.	http://stitch.embl.de/
	SIDER	Database of adverse drug reactions from marketed medicines.	http://sideeffects.embl.de/
	Comparative Toxicogenomics Database (CTD)	Manually curated database of over a million interactions between chemicals and genes and over 1.6 million associations between chemicals and diseases and over 15 million associations between genes and diseases.	http://ctdbase.org
	PharmGKB	Database of drug information including dosing guidelines, drug labels, signaling pathway diagrams, drug-gene associations, and drug-phenotype relationships.	www.pharmgkb.org
	Drug Gene Interaction Database (DGIdb)	Database and web tool for mining over 14,000 drug-gene relationships.	http://dgidb.genome.wustl.edu/
**Drug combinations**	Drug Combination Database (DCDB)	Data from 1363 drug combinations.	www.cls.zju.edu.cn/dcdb/
**Protein-protein interactions**	BioGrid	Database of over 720,000 protein and genetic interactions from model organisms and humans from over 41,000 publications.	http://thebiogrid.org/
	STRING	Database of known and predicted protein interactions, including both direct and functional associations. It currently covers 5,214,234 proteins from 1133 organisms.	http://string-db.org/
**Gene expression data**	Connectivity Map (CMap)	Gene expression profiles from 1309 FDA approved small molecules tested in 5 human cell lines.	www.broadinstitute.org/cmap/
	Gene Expression Omnibus (GEO)	Public repository of gene expression data.	http://www.ncbi.nlm.nih.gov/geo/
**Kinase inhibitors**	K-Map	Web tool that identifies kinase inhibitors for a set of query kinases.	http://tanlab.ucdenver.edu/kMap/
**Pathways**	Reactome	Pathway database with visual representation for 21 organisms, which includes over 1500 human pathways.	www.reactome.org
	KEGG Pathways	Large collection of manually drawn pathway maps of molecular interaction networks for various biological processes.	www.genome.jp/kegg/pathway.html
**Network visualization**	Cytoscape	Open source software platform for network analysis and visualization.	www.cytoscape.org
**Computational modeling**	Netflux	Modeling and simulation tool for construction of normalized-Hill models of signaling networks from user defined species interactions.	http://code.google.com/p/netflux/
	CellNOpt	Free software for creating logic-based models of signaling networks.	www.cellnopt.org
	BioModels Database	Repository of computational models of biological processes. Includes both peer-reviewed models and models produced automatically using pathway resources like KEGG.	www.ebi.ac.uk/biomodels-main/
**Experimental resources**	Cancer Cell Line Encyclopedia	Detailed genetic characterization of ~1000 cancer cell lines.	www.broadinstitute.org/ccle/home
	Genomics of Drug Sensitivity in Cancer (GDSC)	Drug sensitivity data from hundreds of genetically characterized cancer cell lines perturbed with a wide variety of anti-cancer agents Part of an ongoing project to discover therapeutic biomarkers.	www.cancerrxgene.org
	NCI-60 DTP	Drug screen data from a diverse panel of 60 human cancer cell lines with extensive molecular profiling.	http://dtp.nci.nih.gov/index.html
	Cancer Therapeutics Response Portal	Drug sensitivity data of 242 genetically characterized cancer cell lines treated with 354 different small molecule probes and drugs. Each compound selectively targets a distinct part of cell wiring and collectively affect a vast array of cell processes.	www.broadinstitute.org/ctrp

## Quantifying synergistic drug combinations

When presenting the results of drug combination studies, it is important to have a standard to statistically define synergistic drug pairs. Two commonly used methods for quantifying synergy between drug combinations are Loewe additivity and Bliss independence. Loewe additivity is based on the assumption that the two inhibitors act through a similar mechanism while Bliss independence assumes independent mechanisms [17].

### Loewe additivity

Using Loewe additivity, the concentration of two inhibitors (A and B) which alone results in X% inhibition of the target ([*I*_A_]_X%_, [*I*_B_]_X%_) can be used to calculate the theoretical concentrations of each inhibitor needed to achieve the same X% inhibition when combined ([*C*_A_]_X%_, [*C*_B_]_X%_).

1 $$ 1=\frac{{\left[{C}_A\right]}_{X\%}}{{\left[{I}_A\right]}_{X\%}}+\frac{{\left[{C}_B\right]}_{X\%}}{{\left[{I}_B\right]}_{X\%}} $$

**Figure 2:**
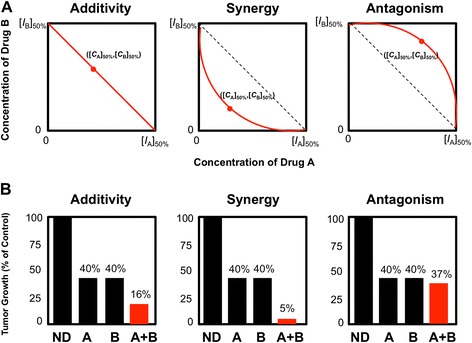
**Examples of Loewe Additivity and Bliss Independence in defining drug interactions. A)** Additivity, synergy and antagonism of drug combination as defined by Loewe Additivity. Let x and y-axes represent concentrations of drugs A and B to achieve a defined effect X% (e.g., X = 50% for half maximal inhibitory concentration (IC_50_) of [*I*_A_]_50%_ and [*I*_B_]_50%_), respectively. The coordinates ([*I*_A_]_50%_,0) and (0, [*I*_B_]_50%_) represent the concentration for drugs A and B, respectively. The line of additivity is constructed by connecting these two points for a 50% effect isobologram plot. The concentrations of the two drugs used in combination to provide the same effect X% (e.g. X = 50%) will be denoted by point ([*C*_A_]_50%_,[*C*_B_]_50%_) and are placed in the same plot. Synergy, additivity, or antagonism will be determined when this point ([*C*_A_]_50%_,[*C*_B_]_50%_) is located below, on, or above the line, respectively. More generally, linear, concave, and convex isoboles represent non-interacting, synergy, and antagonistic drug combination, respectively. **B)** Additivity, synergy and antagonism of drug combination as defined by Bliss Independence. For example, if two non-interacting drugs (A and B) each result in 40% tumor growth compared to control (*E*_A_ = 0.4, *E*_B_ = 0.4), then the predicted tumor growth when combined would be *E*_C_ = (0.4 x 0.4) = 0.16, (16% of control). If the observed combined (A + B, red bar) tumor growth is similar to, less than, or greater than 16% of control, then the combination would be deemed as additive, synergistic, or antagonistic, respectively. N.D. denotes no drug (control).

The Loewe additivity applies the isobologram analysis to evaluate the combination effects of two drugs at a given effect. For example, in a Cartesian coordinate plot where x and y-axes represent concentrations of drugs A and B to achieve a defined effect X% (e.g., X = 50% for half maximal inhibitory concentration (IC_50_) of [*I*_A_]_50%_ and [*I*_B_]_50%_), respectively. The coordinates ([*I*_A_]_50%_,0) and (0, [*I*_B_]_50%_) represent the concentration for drugs A and B, respectively. The line of additivity is constructed by connecting these two points for a 50% effect isobologram plot. The concentrations of the two drugs used in combination to provide the same effect X% (e.g. X = 50%) will be denoted by point ([*C*_A_]_50%_,[*C*_B_]_50%_) and are placed in the same plot. Synergy, additivity, or antagonism will be determined when this point ([*C*_A_]_50%_,[*C*_B_]_50%_) is located below, on, or above the line, respectively. More generally, linear, concave, and convex isoboles represent non-interacting, synergy, and antagonistic drug combination, respectively (Figure 2A).

This approach led to the development of the combination index (CI) popularized by Chou and Talalay [18]. Here, the CI provides a quantitative measure of the extent of drug interaction at a given effect. It measures the combination concentrations of drugs A and B to produce a effect X%, [*C*_A_] and [*C*_B_], normalized by their corresponding concentrations that produces the same effect as a single agent, [*I*_A_] and [*I*_B_], respectively. CI value is calculated by:

2 $$ CI=\frac{\left[{C}_A\right]}{\left[{I}_A\right]}+\frac{\left[{C}_B\right]}{\left[{I}_B\right]} $$

where CI value <1, =1, and >1 represent synergy, additivity, and antagonism, respectively.

### Bliss independence

Bliss independence is based on probability theory and assumes the two inhibitors are working through independent mechanisms [15]. The inhibitors do not interfere with each other, but contribute to a common result. Unlike Loewe additivity, calculating Bliss independence does not require determination of dose–response curves for the individual compounds to determine the theoretical results, making it easier to compute [19]. Bliss independence models the combined effect (*E*_T_) as the product of the individual effects with drugs A (*E*_A_) and B (*E*_B_). The predicted combined effect (*E*_T_) is computed by:

3 $$ {E}_T={E}_A\times {E}_{\mathbf{B}} $$

where each effect (*E*) is expressed as fractional activity compared to control between 0 (100% inhibition) and 1 (0% inhibition). For example, if drug A and drug B each result in 40% tumor growth compared to control, then the predicted tumor growth when combined would be (0.4)*(0.4)*(100%) = 16% of control according to Bliss Independence. The predicted combined inhibition level would therefore be 100%-16% = 84% inhibition of tumor growth. If the actual tumor growth when drug A and B are combined is less than 16% of control (greater than 84% growth inhibition), then the compounds would be synergistic by Bliss Independence. If the tumor growth level is greater than 16% of control (less than 84% growth inhibition), then the compounds would be defined as antagonistic (Figure 2B).

These two methods produce different results, and it is uncertain which method performs better with uncertainty of mechanism and noisy data [17,20]. Drugs inhibiting parts of the same linear pathway may act according to Loewe additivity [17]. Drugs nonexclusively affecting parallel pathways may act according to Bliss independence. Experimental characterization of drug combinations typically involves generating dose response curves with the inhibitors separately and combined. The experimental dose response curve data can then be compared to the predictions of Loewe additivity or Bliss independence to determine if the drugs are acting synergistically.

## Computational models of signaling networks

Given the complexity of the signaling networks controlling systemic diseases such as cancer, computational models of cell signaling pathways are important tools for increasing understanding of pathological signaling and prioritizing targets to test experimentally [21]. Models can be used to quantify systems properties that are often not apparent in individual experiments. Through model simulations, one can predict the relative importance of various proteins in the network, the presence of signal amplification, and the role of feedback and cross-talk [22]. These features will be important in the prediction of viable drug combinations. While model predictions require experimental validation, they are useful tools for prioritizing targets for experimental planning.

### Mass action and enzyme kinetics-based models

Three predominant signaling network modeling approaches are mass action and enzyme kinetics-based, logic-based, and statistical association-based models. Mass action models are biochemically detailed kinetic models that typically represent interactions between molecular species in the signaling network as ordinary differential equations (ODEs) and require selection of parameter values for concentrations of species in the network and rate constants controlling protein-protein associations [23]. Many of these parameters may be unavailable in the literature and can either be measured experimentally or fit to the data by minimizing an objective function such as the sum of squared error.

When training parameters to data, it is important to determine the importance of parameter selection on model predictions. For example, Chen et al. measured parameter sensitivity for several independent fits and saw that the rank order of the most sensitive parameters was nearly the same across the fits for a given output, therefore parameter uncertainty did not affect major model predictions [24]. In another approach, Iadevaia et al. developed a mass-action model of the IGF-1 signaling network in a breast cancer cell line with 161 unknown parameters and fit the model to the time courses of six proteins measured with reverse-phase protein array [25]. Given the uncertainty in parameter estimation with so many unknown parameters, they identified ten sets of parameters using particle swarm optimization that equally fit the experimental data. Model predictions were averaged from three randomly sampled sets of the ten parameter sets. The trained model was then used to identify beneficial drug combinations in a breast cancer cell line.

Mass-action network models have been used to predict new beneficial drug combinations for cancer. As an example, Faratian et al. used a mass-action model of heregulin-induced HER2/3 signaling through MAPK and PI3K to study the role of PIK3CA activation in Receptor Tyrosine Kinase (RTK) inhibitor resistance [26]. Model results demonstrated that the ratio of PTEN to activated PIK3CA predicted resistance to RTK inhibitors. This finding could therefore be used to predict patient response to anti-HER2 therapies based on clinical measurements of PTEN. It predicts that PIK3CA inhibition should be paired with RTK inhibitors in patients with tumors with low PTEN, a negative regulator of PI3K signaling. Another group developed a mass action kinetics model of PI3K signaling by ERBB receptors including ligand binding, dimerization, internalization, recycling, and degradation [27]. Sensitivity analysis of this model predicted an important role of ERBB3 in AKT activation, which was then validated in mice xenografts. Sensitivity analysis could be used in future work to find drug combinations that may work synergistically with ERBB3 inhibition.

### Logic-based models

A limitation of mass-action modeling approaches is the amount of data required to generate specific values for the abundance and rate constant parameters, which can be prohibitive for large scale network reconstructions (Figure 1) [28]. Logic-based models use network topology without the need for specific parameter values. Network interactions are modeled with OR, AND, and NOT Boolean logic gates. Each species in the network takes a value of 0 (inactive) or 1 (active) based on the state of its effectors [29]. As an example, Sahin et al. developed a Boolean model of ERBB signaling of G1/S cell cycle transition [30]. The group used computational knockouts of network proteins, validation experiments with RNAi, and model revision based on proteomic data, to predict the effects of combined inhibition of ERBB2 and c-MYC or EGFR. A combination therapy targeting c-MYC and ERBB2 was predicted to improve treatment for breast cancer that is *de novo* resistant to ERBB2 inhibition. Another group developed a Boolean logic model of apoptosis signaling in Leukemic T-Cell large granular lymphocytes [31]. The authors used the model to determine species that controlled apoptosis and experimentally validated two of these species, sphingosine kinase 1 and NFκB. Given the limitations in representing species as either on or off, this modeling approach has been extended to accommodate intermediate activity states using fuzzy logic [32].

### Normalized hill differential equation modeling approach

While logic-based modeling approaches benefit from simple construction using network topology, results can be difficult to interpret due to assignment of discrete values to continuous variable such as concentration of active species, sensitivity to temporal node-updating schemes, and incompatibility with many systems analysis tools such as quantitative sensitivity analysis [33]. To address the limitations of mass-action and logic-based models, Kraeutler et al. developed the normalized Hill differential equation modeling approach, which uses logic-based differential equations to represent activation or inhibition by molecular species in the network [33]. Cross-talk is represented with AND and OR gates and species activation is continuous over time and in units of fractional activation instead of concentration. Therefore protein abundance parameters are not required like with mass-action models. Interactions between species in the network are modeled with normalized Hill equations with 3 parameters: reaction weight, half maximal effective concentration (EC_50_), and Hill coefficient. While these parameters can be fit to data, using default values generated highly similar quantitative predictions as a previously constructed detailed biochemical model of the same pathway which used 88 parameters from literature [33,34]. Therefore, this approach allows for straightforward model construction of a known network topology even if kinetic and abundance parameters are unknown, like with logic-based modeling, while also allowing for prediction of dynamics and systems analysis tools such as quantitative sensitivity analysis.

The normalized-Hill modeling approach is a valuable tool for model construction of larger networks with more unknown parameters. As an example, Ryall et al. used this approach to model the cardiac hypertrophy signaling network, which contained 106 species and 193 reactions [35]. Since cardiac myocytes have minimal capacity for proliferation, many of these pathways also regulate proliferation in cancer cells [36]. Quantitative systems analysis revealed the most prevalent species involved in growth of cardiac myocytes, prioritizing future experimental targets [35]. While Ras, the largest signaling hub, was the highest influencer on cell size, the correlation between the number of connections a species has and its influence was low. Moreover, highly influential species were at many levels in the network, not just close to the output level. These findings demonstrate the need for model reconstructions to predict important drug targets in cell signaling networks. Highly influential species are not obvious from intuition alone or data from gain or loss of function studies of single genes [37].

Ryall et al.’s analysis of the hypertrophy signaling network also looked at the presence of different signaling motifs such as bi-fan and feed-forward loops. Motifs can affect network properties such as signal filtering, acceleration, pulse generation, ultra-sensitivity, stability, and robustness [38-40]. Yin et al. modeled three-node enzymatic networks with many different topologies to study the effect of topology on drug combinations [41]. Model simulations were conducted to identify motifs that could result in synergy. Most of the combinations were not dependent on parameter selection, demonstrating that network topology can be used to predict synergistic combinations. Moreover, synergistic drug combinations were found in both parallel and series drug combinations. In a similar study, Zhang et al. made reduced models of the convergence of two signaling pathways on a target and observed synergy in only a subset of the motifs [42]. Synergy had a greater likelihood in motifs with negative feedback between the target and an upstream effector or mutual inhibition between parallel signaling pathways. These findings suggest that searching for synergistic motifs within a cancer signaling network topology can be a useful strategy in prioritizing drug combinations to test experimentally. Networks exported into Cytoscape [43], a open source software platform for network visualization, can use the Netmatch plug-in [44] to quickly search for motifs of interest.

### Statistical association-based modeling approach

Network modeling approaches are useful when network topology is known, but these approaches can be biased towards established pathways and may miss novel interactions. Statistical association-based models do not depend on prior knowledge of pathways and instead use correlations and patterns in experimental data to predict network structure. As an example, Ryall et al. used data of correlations among cell shape features and expression of 12 genes relevant to cardiac hypertrophy to identify a network map linking input modules to output modules [45]. Drug combinations could then be prioritized by selecting targets that enabled adaptive module signaling and prevented maladaptive module signaling. Molinelli et al. developed a network inference algorithm based on Belief Propagation [46] to construct networks from phenotypic screen data [47]. They applied their method to screen data from a melanoma cell line and identified both new and established pathway interactions and then used the network to predict efficacious drug targets. Another useful approach for network inference is Bayesian network computational methods [48].

## Signature-based approaches for predicting drug combinations

Many effective drug combinations have been discovered using correlations in gene expression signatures. One useful tool for this is the Connectivity Map (CMap) database [49]. The first-generation CMap contained gene expression profiles from three cancer cell lines perturbed by 164 distinct small-molecule compounds. The second generation of CMap (CMap 2.0) includes gene expression profiles from 1,309 small molecules including FDA approved drugs tested in five human cancer cell lines [49,50]. This method assumes gene expression changes can be used as a “universal language” to connect distinct biological states (e.g. diseases), allowing for the successful repurposing of compounds [51,52]. In short, drugs known to be effective in one disease can serve as candidates for use in other diseases marked by similar gene expression changes. Users query the database to compute similarity metrics between a test gene expression signature and each reference set. Similarity metrics are scaled between −1 and +1, where a positive score indicates positive correlation and a negative score indicates negative correlation. An advantage of this approach is the ability to query CMap with publicly available gene expression data from sources such as Gene Expression Omnibus (GEO) [53], therefore facilitating rapid drug combinations prediction for experimental validation [54].

As an example, Riedel et al. applied CMap to predict drugs that would prevent resistance to chemotherapy agents in lung cancer cell lines [55]. Genes with the highest changes after treatment with docetaxel were analyzed using CMap to identify drugs with negative connectivity scores, indicating these drugs had antagonistic effects on the genes associated with docetaxel resistance. PI3K inhibitor LY294002, which was highly ranked among these antagonistic compounds, was tested *in vitro* with docetaxel and found to synergistically increase cytotoxicity. Wei et al. used a similar approach to predict drugs to overcome resistance to glucocorticoid treatment in acute lymphoblastic leukemia [52]. Microarrays of pre-treated cell lines either sensitive or resistant to glucocorticoid *in vitro* were used to define a sensitive/resistant gene signature. Using CMap, mTOR inhibitor rapamycin was found to induce a highly similar signature, leading to the hypothesis that mTOR inhibition could induce glucocorticoid sensitivity. Follow-up experiments supported this hypothesis and showed that rapamycin conferred sensitivity through down-regulation of MCL1. Therefore, CMap is a useful tool for using gene signatures to predict drug combinations that may overcome drug resistance.

Inspired by the CMap concept, Kim et al. recently developed K-Map (Kinase Inhibitor Connectivity Map) that systematically connects a set of query kinases to kinase inhibitors based on quantitative profiles of the kinase inhibitor activities [56]. Instead of gene expression signatures, Kim et al. used the kinase activity profiles as the “language” for connecting kinases and small molecules in K-Map to reveal the complex interactions of kinases and inhibitors. By querying K-Map with the essential kinases mediating resistance to EGFR-inhibitor gefitinib in an EGFR mutant non-small cell lung cancer (NSCLC) cell line, bosutinib was predicted to be a more effective drug for killing EGFR mutant cancer cells. Follow up *in vitro* experiments confirmed that bosutinib alone is a more effective agent than gefitinib, and that the combination of bosutinib and gefitinib had synergistic effects in EGFR mutant NSCLC cells [57]. This demonstrates the utility of K-Map in connecting kinases with kinase inhibitors and suggesting candidates for drug combinations.

## Network-based approaches for predicting drug combinations

Other computational approaches have been developed to predict drug combinations using data from high-throughput screens and drug databases. Pal and Berlow developed an algorithm based on set theory that uses tumor drug sensitivities and kinase inhibition profiles for a set of individual drugs to predict the tumor sensitivity to new drugs or drug combinations [58]. The algorithm applies the following rules to generate circuit representations of tumor pathways: 1) drugs that inhibit a superset of an effective set of inhibited kinases will also be successful in inhibiting tumor growth and 2) drugs inhibiting subsets of ineffective sets of inhibited kinases will also be unsuccessful. These circuits reveal a set of kinases that are most predictive of drug sensitivity and depict combinations of kinases that need to be inhibited to prevent tumor growth. This analysis is helpful for identifying drug combinations that inhibit a minimal set of kinases with as few off target effects as possible to minimize negative side effects. This approach was validated using data from four canine cancer cell lines given 60 different drugs at four different concentrations to generate IC_50_ values [59]. Tang et al. expanded on this algorithm to improve the computational cost and accuracy with drug screen data with little overlap between drug target profiles [60]. While this approach requires a lot of experimental data from drug screens to be useful (Figure 1), technological advancements are enabling larger drug screens at lower costs.

Given the high cost of exhaustive drug screens, Gujral et al. exploited the polypharmacology of kinase inhibitors by developing an approach to select the most predictive kinase targets from a smaller scale drug screen of multi-target drugs [61]. They performed a phenotypic screen to identify kinases regulating cell migration using an optimal set of 32 kinase inhibitors. Elastic net regularization was then used to deconvolute the polypharmacology of the kinase inhibitors, identifying kinases with the greatest explanatory power for the phenotype. Elastic net regularization regresses an output variable against a set of predictor variables (kinase activity) and invokes a penalty on the number of variables in order to eliminate kinases with insignificant contributions. This is a useful approach for reducing experimental time and cost by extracting more information from a smaller set of compounds in drug screens.

Huang et al. used drug genomic profiles from the Connectivity Map database to construct a drug functional network and then grouped drugs into modules with similar transcriptional responses [62]. They then built disease-signaling networks highlighting defective signaling modules based on patient genomic profiles and protein interaction data. The DrugComboRanker algorithm ranked potential drug combinations by selecting drugs with high overlap in the disease network, affecting multiple key signaling modules. In a similar approach, Pang et al. developed an algorithm to identify combination therapies by building a network of drug-target interactions from the DrugBank database [63]. Given an input disease gene set, the algorithm selected drugs that maximized on target effects and minimized off target effects. This algorithm also identifies drug combinations of more than two drugs, which would be unfeasible to predict using a high-throughput screen. These approaches allow researchers to take advantage of publicly available drug data to prioritize combinations for experimental validation.

Moreover, Liu et al. have developed a database of both successful and unsuccessful drug combinations (DCDB) [64]. The current version of the database contains 1,363 drug combinations involving 904 individual drugs and 805 targets. The database provides information about the potential mechanism, drug interactions, indication, published study, development status, and targets. The ability to analyze patterns in successful and unsuccessful combinations with this database will be useful for systems analysis of drug combinations and rational experimental design. As an example, Xu et al. constructed a network of successful drug interactions using data from the Drug Combination Database [65]. Analysis revealed that drugs with similar therapeutic effects tended to cluster together in the network and targets of hub drugs were often membrane or membrane-associated proteins. They used these observations to develop a statistical approach to predict new drug combinations.

Cheng et al. constructed a global human kinome interaction map by integrating kinase-substrate interactions, kinase-drug interactions, protein-protein interactions, and atomic resolution three-dimensional structural protein-protein interactions [66]. Their analysis of the topological features of these networks revealed an enrichment of hubs as drug targets. While targeting hubs can be beneficial due to cascading effects, their analysis revealed that targeting hubs also increases risk of adverse drug reactions and drug resistance through feedback and crosstalk.

Network based approaches have also been used to study drug-drug interactions. As an example, Cheng and Zhao developed a comprehensive drug-drug interaction network incorporating 6946 interactions of 721 approved drugs using data from DrugBank [66]. They then calculated drug-drug pair similarities using four features: phenotypic similarity, therapeutic similarity, chemical structure similarity, and genomic similarity. They applied five machine learning-based models to the dataset to predict drug-drug interaction, with the overall hypothesis that drugs with similar chemical structure, target proteins, adverse drug reactions, and therapeutic purposes have high probability of drug-drug interaction. They tested the model on antipsychotic drug-drug interactions and found literature support for predictions of drug-drug interactions involving weight gain and P450 inhibition. This approach demonstrates the power of harnessing network-based drug-drug interactions to reveal new information on adverse drug effects and provide additional filtering rules for drug combination studies.

## Integrating functional genomics and computational methods for identifying drug combinations

Large scale knockdown screens using RNA interference (RNAi) can also be used to identify potential drug combinations [67]. RNAi screens can identify genes that lead to sensitivity or resistance to a drug of interest. As an example, an RNAi screen conducted by Berns et al. showed that knockdown of PTEN decreased sensitivity to trastuzumab in BT-474 breast cancer cells [68]. Follow-up studies showed that assessment of both the loss of PTEN expression and activating mutations in PIK3CA could predict the risk for HER2 amplified tumor progression. Drugs reducing PI3K signaling may therefore increase response to trastuzumab. In another study, Prahallad et al. used an RNAi screen to identify kinases whose knockdown synergized with BRAF(V600E) inhibition in colon tumors [69]. Follow-up experiments demonstrated synergy between cetuximab (EGFR inhibitor) and vemurafenib (BRAF inhibitor). The rational combination of cetuximab and vemurafenib is currently being evaluated in clinical trials.

Pritchard et al. used RNAi signatures of eight cell death genes to determine the mechanism of drug combination effects in lymphoma cells [70]. Single drugs were classified based on their similarity to the RNAi signatures of well-characterized compounds with known mechanisms. They then generated signatures for drug combinations to see if the signature was more similar to results from one of the drugs alone, an average of the two, or a unique signature. Results showed that the combination signature was usually a weighted composite of single drug effects where one drug potentiated the mechanism of the other or the two drugs produced an additive effect. Interestingly, they observed that applying larger pools of drugs to tumors reduced the genetic heterogeneity, which could be prohibitive in selection of personalized drug treatments for patients based on biomarkers.

Spreafico et al. identified non-canonical Wnt pathway mediated resistance to MEK1/2 inhibitor Selumetinib in colorectal cancer cells by integrating gene set enrichment analysis and synthetic lethality screens [71]. Using cyclosporine A (CsA) as a drug to inhibit non-canonical Wnt pathway, they validated that the combination of Selumetinib and CsA has synergistic anti-proliferative effects both *in vitro* and *in vivo* patient-derived xenografts. This rational combination is now being translated into a Phase I clinical study (ClinicalTrials.gov ID: NCT02188264). This illustrates the utility of integrating functional genomics screens with bioinformatics to identify and translate drug combinations into clinical study.

## High-throughput drug combination screens

While the number of FDA approved drugs makes exhaustive drug screens unfeasible (Figure 1), there have been efforts to reduce the search space in an unbiased manner. Tan et al. used pools of ten drugs in 384-well plates to study all possible pairs of 1,000 compounds in the minimum number of wells possible in order to find drugs combinations that synergistically prevent HIV replication [72]. Synergistic wells from the primary screen are then deconvolved into possible synergistic pairs for a secondary screen. Results revealed an enrichment of anti-inflammatory drugs in the synergistic pairs.

Roller et al. conducted a functional chemical genetic screen of 300 drug combinations in nine melanoma cell lines and identified pairs of compounds that synergistic increase cytotoxicity [73]. Interestingly, the synergistic cytotoxicities identified did not correlate with the known oncogene RAS and RAF mutational status of the melanoma cell lines. From this screen, they identified sorafenib (a multi-kinase inhibitor) and diclofenac (a non-steroidal anti-inflammatory drug) to be the most robust drug combination that has synergistic effects across the melanoma cell lines. By using this functional chemical genetic screen, the authors uncovered novel interactions between signaling inhibitors that would not be predicted based on current understanding of the signaling networks. Their results suggest that the underlying signaling networks controlling drug responses can vary substantially based on unidentified elements of cell genotype. In another study, Griner et al. conducted a large scale screen of multiple concentrations of 500 compounds with ibrutinib in activated B-cell-like diffuse large lymphoma cells [74]. They discovered many compounds that interacted favorably with ibrutinib, including inhibitors of PI3K signaling, the Bcl2-family, and the B-cell receptor pathway.

One of the major ongoing initiatives at the Developmental Therapeutics Program of the National Cancer Institute, U.S. National Institute of Health, is the large-scale high-throughput drug combination screening of 100 FDA approved drugs. The first set of screening results generated 5,000 possible drug combinations in the NCI-60 cancer cell lines panel [75]. The goal of this project is to identify novel drug combinations that are more active than the single agents alone. As all the drugs tested have been FDA approved, any drug combinations identified from this screen may rapidly translate into the clinic. As the NCI-60 cell lines panel have been fully characterized by various “omics” technologies, the release of this drug combination matrix to the public could facilitate the development of novel computational methods to integrate, predict, and mine the interactions between molecular markers and drug combinations.

While substantial intratumoral heterogeneity has been detected in cancer patients using next generation sequencing technologies [76], current drug combination prediction methods have primarily focused on targeting the predominant tumor subpopulation. To study the effect of different tumor subpopulations on treatment efficacy, Zhao et al. developed a multi-objective linear optimization algorithm to select optimal drug combinations for heterogeneous tumors by maximizing efficacy and minimizing toxicity [77]. Their goal was to determine the best drug combinations to minimize all subpopulations. They experimentally validated the algorithm’s prediction of two-drug combinations with three-component heterogeneous tumors created using RNA interference [78]. They then expanded the model to simulate more complex tumors and greater numbers of drugs [77]. Their results revealed that intratumor heterogeneity influences the prediction of effective drug combinations. Different predictions are made depending on if all tumor subpopulations are considered or just the predominant subpopulation. This approach represents a step forward of predicting drug combinations to tackle tumor heterogeneity in the era of precision oncology.

## Perspective

Given the experimental costs of exhaustively testing drug combinations, computational models of signaling networks will be especially useful in pre-clinical screening of combinations of compounds. Model simulations reveal non-intuitive effects of drug combinations [17]. Due to the size and complexity of cancer signaling, modeling strategies accommodating reconstruction of larger networks while still being compatible with quantitative systems analysis tools will be especially useful [79]. Global network models allow for more comprehensive and unbiased discovery of therapeutic targets than experimental approaches based on *a priori* selection of important pathways [16]. One of these methods is the recently described normalized-Hill modeling approach, which even with minimal biochemical data from literature enables a global view of quantitative functional relationships between every node in a network [33]. This method was used to identify the most important nodes in a integrative network model of cardiac hypertrophy with 106 nodes and 193 reactions and default parameter values [35]. Many of these cardiac hypertrophy signaling pathways also play important roles in tumor growth. While model predictions need to be experimentally validated, models can substantially improve hypothesis generation and experimental planning.

**Figure 3:**
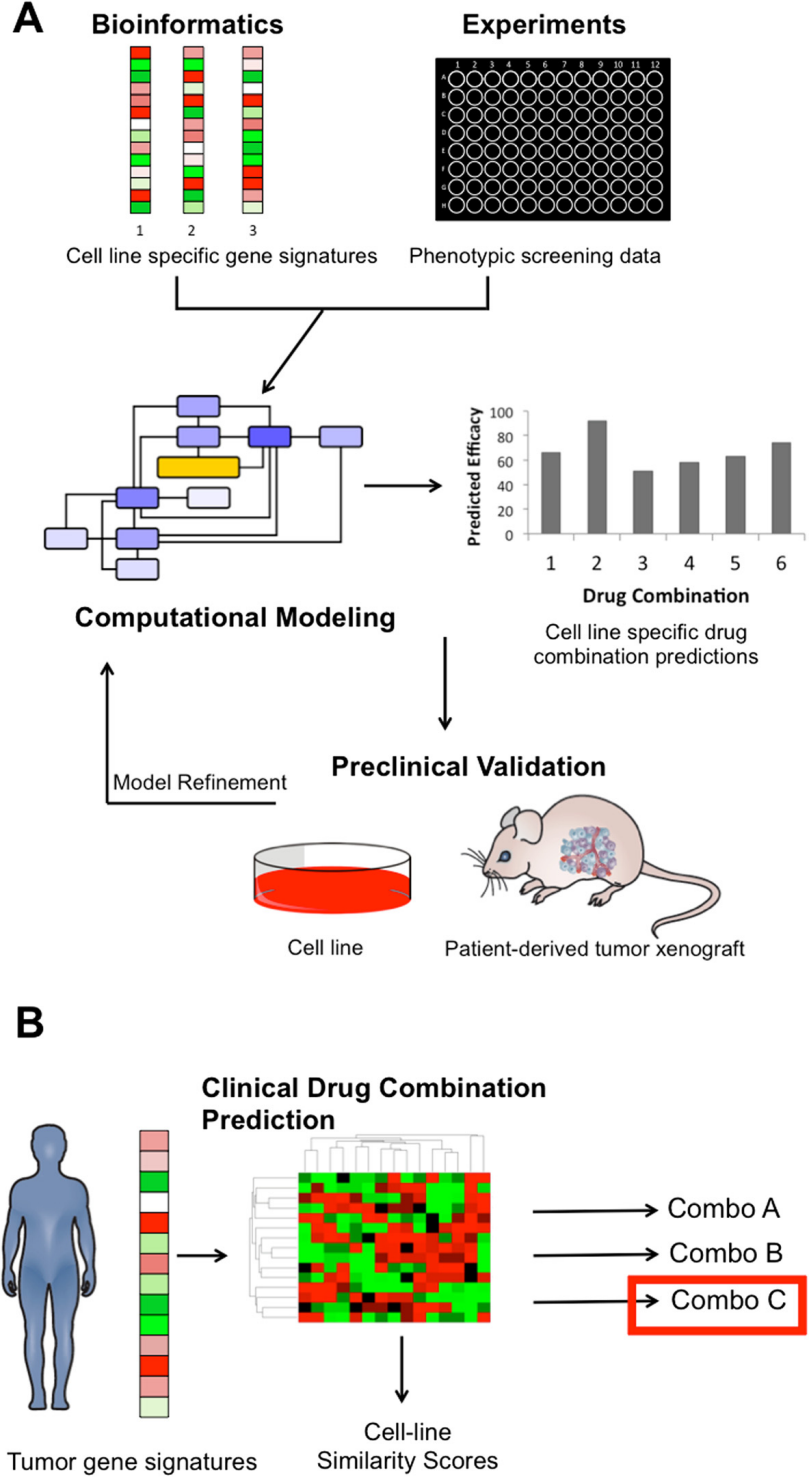
**Future strategy for drug combination predictions with parallel integration of computational modeling, preclinical testing, and clinical trials. A)** Future combinatorial drug discovery approaches will benefit from tighter integration of gene signatures and phenotypic screen data with computational models, tuning the models to specific cancer cell-lines. Model simulations enable prediction of effective drug combinations for preclinical validation. Preclinical data can then be used to further refine computational models. **B)** For clinical application, patient gene signatures can be clustered with gene expression signatures from previously modeled cell lines. Similarity scores can then be computed to find the most similar model to the patient’s tumor for selection of the appropriate drug combination.

In addition to larger network reconstructions, future modeling efforts will benefit from tighter integration of high-throughput sequencing, proteomics, and phenotypic screen data (Figure 3A). This will enable tuning of a model to an individual patient’s tumor, which would be beneficial for use in personalized medicine. Comprehensive double knockdown model simulations would enable personalized prediction of drug combinations for patients. The ability to readily adapt models to different situations is important in cancer research since the molecular networks are not fixed within a particular cancer type [79]. Patients that share the same mutation and tumor type can have different responses to a drug [80]. Genetic background, cell lineage, and exogenous signals can influence the network behavior [79]. Efficacy data identified from *in vitro* and *in vivo* experiments would then be used for model refinement so more informed predictions of drug combinations can be made in future studies.

Predicted drug combinations should be validated in cancer cell lines and in relevant *in vivo* human disease models such as patient-derived tumor xenografts [81]. These models, however, typically overestimate the clinical benefit due to factors such as tumor heterogeneity, differences in tumor microenvironment, and inaccurate estimates of drug exposure [82]. Therefore, it is important to have a high threshold when choosing effective combinations, ignoring modest inhibitions of tumor growth in favor of combinations promoting cancer cell death and tumor regression.

Another opportunity for improved design of combination therapies is through quantitative systems pharmacology approaches integrating cell signaling network models with pharmacokinetic-pharmacodynamic (PK/PD) models [83]. Quantitative systems pharmacology uses multi-scale data to better understand and ultimately predict how drugs affect cellular networks and human pathophysiology [84]. Mechanistic models of cell signaling networks are linked to PK/PD models of physiological processes at the level of tissues and organisms. These models will enable patient-specific prediction of therapeutic and toxic drug responses and drug resistance mechanisms, improve translation of *in vitro* discoveries to patients, and enhance discovery of pharmacodynamic biomarkers.

Additionally, future work will benefit from parallel integration of computational modeling, preclinical testing, and clinical trials, where data from each approach can be used for refinement of the other. Computational models tuned to specific cancer cell lines using bioinformatics and experimental data could be perturbed to make predictions of effective drug combinations to validate in preclinical models (Figure 3A). For clinical application, similarity scores between patients and previously modeled cell lines could be calculated using statistical clustering (Figure 3B). Drug combinations predicted using the most similar model could then be applied in the clinic.

## Conclusion

It is becoming increasingly apparent that drug combinations will be essential for improving therapies for complex diseases such as cancer [19]. The signaling pathways controlling these systemic diseases are highly interconnected, with cross-talk, redundancy, and feedback, making single-target therapies much less effective [85]. While combination therapies have the potential to prevent the development of resistance seen in many single drug therapies, it is prohibitively expensive to experimentally test every potential combination, especially when considering combinations of more than two drugs. Here, we highlighted a variety of systems biology applications for advancing the prediction of effective drug combinations, as summarized in Table 2. These methods include computational modeling, gene signature analysis, functional genomics, and high-throughput drug combination screening. Utilization and integration of these systems biology approaches hold great promise in speeding up the development of clinically relevant drug combinations.

**Table 2:** **Summaries of reviewed systems approaches for identifying drug combinations**

**Disease models**	**Method**	**Key findings**	**Validation**	**Reference**
**Computational models of cell signaling networks**				
Breast cancer	Mass-action model	Combined inhibition of MEK and PI3K optimally decreased cell viability.	*in vitro*	[25]
Ovarian cancer	Mass-action model	the ratio of PTEN to activated PI3K predicts RTK inhibitor resistance	*in vitro*	[26]
Ovarian cancer	Mass-action model	ErbB3 inhibition inhibits the ErbB-PI3K network more potently than current therapies.	*in vivo (rodent)*	[27]
Breast cancer	Logic-based	Combined inhibition of c-MYC and ERBB2 improved treatment for trastuzumab resistant breast cancer.	*in vitro*	[30]
T cell large granular lymphocyte leukemia	Logic-based	Sphingosine kinase 1 and NFKB are essential for survival of leukemic T cell large granular lymphocytes.	*in vitro*	[31]
Colorectal cancer	Fuzzy Logic	MK2 and MEK are co-regulators of ERK and EGF induced IKK inhibition.	*in vitro*	[32]
Cardiac hypertrophy	Normalized-Hill model	Ras had the greatest influence on hypertrophy and correlation between node degree and influence is low.	*in vitro*	[35]
Various	3-node enzymatic models	Identified consistent synergistic and antagonistic motifs.	*in silico*	[41]
Various	4-node enzymatic models	Synergy is more prevalent in motifs with negative feedback between the target and an upstream effector or mutual inhibition between parallel pathways.	*in silico*	[42]
Cardiac hypertrophy	Statistical association model	Maladaptive and adaptive hypertrophy features were in separate modules in the simplified hypertrophy network map generated by k-means clustering of ligands and phenotypic outputs.	*in vitro*	[45]
Melanoma	Statistical association model	PLK1 inhibition increases cytotoxicity of RAF inhibitor resistant melanoma cells.	*in vitro*	[47]
Various	Statistical association model	Reconstructed classic T cell signaling network using multiparameter single-cell data and Bayesian network inference.	*in vitro*	[48]
**Signature-based approaches**				
Lung cancer	CMap	PI3K inhibition enhanced docetaxel-induced cytotoxicity	*in vitro*	[55]
Lymphoblastic Leukemia	CMap	mTor inhibition induced glucocorticoid sensitivity by decreasing MCL1	*in vitro*	[52]
Lung cancer	K-Map	The combination of bosutinib and gefitinib has synergistic effects in EGFR mutant non-small cell lung cancer	*in vitro*	[57]
**Network-based approaches**				
Osteosarcoma	Target Inhibition Map (TIM)	Developed an algorithm using a training set of drug sensitivities with known targets to predict responses to new drugs and combinations.	*in vitro*	[58,59]
Breast and pancreatic cancer	TIMMA	Target Inhibition inference using Maximization and Minimization Averaging (TIMMA). Improved computational cost and accuracy of the above TIM approach. Predicted kinase pairs that could be inhibited to prevent cancer survival.	*in vitro*	[60]
Various	Elastic Net Regularization	Performed phenotypic screen using an optimal set of 32 kinase inhibitors. They used an elastic net regulatization algorithm to deconvolute the polypharmacology and identify key kinases regulating cell migration.	*in vitro*	[61]
Lung and breast cancer	DrugComboRanker	Created drug and disease functional networks based on genomic profiles and interactome data. Drug combinations are predicted by identifying drugs whose targets are enriched in the disease network.	Literature support	[62]
Various	Mixed integer linear programming	Built a network of drug-target interactions from DrugBank. Given an input gene set, the algorithm selects drug combinations that maximize on target effects and minimize off target effects	Literature support	[63]
Various	Systems analysis of Drug Combinations	Drugs with similar therapeutic effects cluster together in a network of successful drug combinations produced using the Drug Combination Database [59]. Network observations were used to develop a statistical approach for predicting drug combinations (DCPred)	Literature support	[65]
Drug-drug interactions	Drug-drug interaction network	Applied five machine learning models to a data set of drug-drug pair similarities including 721 approved drugs to predict drug-drug interactions.	Literature support	[66]
**Integration of functional genomics and computational methods**				
Breast cancer	RNAi screen	PTEN downregulation with active PI3K signaling induce trastuzumab resistance	*in vitro*	[68]
Colorectal cancer	RNAi screen	EGFR inhibition synergizes with BRAF(V600E) inhibition	*in vivo (rodent)*	[69]
Lymphoma	8-gene RNAi signature	Drug combination signatures were usually a weighted composite of single drug effects	*in vitro*	[70]
Colorectal cancer	RNAi screen	The combination of Selumetinib (MEK1/2 inhibitor) and CsA (Wnt inhibitor) has synergistic anti-proliferative effects	*in vivo (rodent)*	[71]
**High-throughput drug combination screens**				
HIV	Pooled screen	Used pools of 10 drugs in 384-well plates to study all possibly pairs of 1000 compounds in the minimum number of wells possible	*in vitro*	[72]
Melanoma	Drug combination screen	Sorafenib (a multi-kinase inhibitor) and diclofenac (NSAID) had synergistic effects across all nine tested melanoma cell lines.	*in vitro*	[73]
Lymphoma	Drug combination screen	Screen of 500 compounds with ibrutinib revealed favorable combinations with inhibitors of PI3K signaling, the Bcl2 family, and B-cell receptor pathway	*in vitro*	[74]
Various cancers	Drug combination screen	Screen of 5,000 combinations of FDA-approved drugs in the NCI-60 cancer cell line panel.	*in vitro*	[75]
Lymphoma	RNAi-modeled tumor heterogeneity	Intatumor heterogeneity influences the prediction of effective drug combinations.	*in vivo (rodent)*	[77,78]
